# Intraocular Scattering, Blinking Rate, and Tear Film Osmolarity After Exposure to Environmental Stress

**DOI:** 10.1167/tvst.10.9.12

**Published:** 2021-08-11

**Authors:** Juan Tabernero, Nery Garcia-Porta, Pablo Artal, Shahina Pardhan

**Affiliations:** 1 Vision and Eye Research Institute, Anglia Ruskin University, Cambridge, UK; 2 Departamento de Electromagnetismo y Electrónica, Universidad de Murcia, Spain; 3 Vision and Hearing Sciences Research Centre, Anglia Ruskin University, Cambridge, UK; 4 Laboratorio de Óptica, Universidad de Murcia, Murcia, Spain

**Keywords:** human eye, light scattering, blinking ratio, tear film, dry environments

## Abstract

**Purpose:**

Dry environments, such as those in offices or aircraft cabins, can potentially generate ocular discomfort and alter the tear film. We compare light scatter, blinking rate, and tear osmolarity in young and older subjects after exposure to low humidity using a controlled environmental chamber.

**Methods:**

Two groups of healthy subjects were recruited; younger (*N* = 13, 27 ± 6 years) and older (*N* = 23, 71 ± 7 years). Measurements were carried out before and after 90-minute exposure to low relative humidity (5%) and constant temperature (23 degrees). Ocular light scatter was measured using a double-pass instrument (OQAS, Visiometrics, Spain). Blinking rate was monitored using an infrared video camera. Tear osmolarity was measured using the TearLab system (Escondido, CA, USA).

**Results:**

Ocular light scatter increased by a factor of 10% after exposure to low humidity in the older group (*P* = 0.03) but did not change significantly in the younger group. Blinking rate increased significantly (40% more blinks) in both groups but there was no difference between the groups. No significant differences in osmolarity were shown between two age groups or as result of environmental stress.

**Conclusions:**

Exposure to dry environment increased light scatter in older subjects. Although more blinks were triggered in both younger and older groups to prevent corneal dehydration, there was no difference between the groups. Blink rate and osmolarity are not associated with the difference in light scatter.

**Translational Relevance:**

Our work approaches a clinical care problem using basic research methods (measuring ocular scatter and blink ratio).

## Introduction

Intraocular scattering limits the quality of vision. Unlike refractive errors and aberrations, intraocular scattering is generated from deflection and diffraction of light by small particles and irregularities present in the ocular media.^1^^–^^3^ Whereas refractive errors and aberrations only deteriorate the central part of the point spread function (PSF) usually limited to within one degree of central vision, light scattering affects areas well beyond this. The effect of light scattering on retinal image quality (“stray light”) is usually perceived as a loss of contrast of the retinal image.^4^^–^^6^

Intraocular scattering can be generated by any parts of the ocular media (i.e., the cornea,^1^ the crystalline lens,^7^ deeper retinal layers^8^ and even sclera).^1^^,^^9^ In a normal, nonpathological population, intraocular scattering is influenced by both genetic and environmental factors.^10^ Aging as well as various pathological conditions, such as cataracts^11^^–^^15^ and dry eye disease^16^^–^^19^ (DED), can affect ocular media and thus increase intraocular scattering. In addition, as the tear film breaks as part of the normal blinking pattern, light scatter increases which in turn decreases contrast sensitivity temporarily until a new blink reconstructs the tear film.^20^^,^^21^

External environmental conditions, such as high temperature, low humidity, and strong winds, present physiological challenges for the maintenance of a healthy tear film. It has been shown that exposure to low humidity alters several important parameters of the tear film, including the evaporation rate, noninvasive tear break-up time, and tear production.^22^^–^^24^ In addition, on a molecular level, inflammatory cytokines and growth factors in tears are altered by exposure to dry environments.^24^^,^^25^ Environmental conditions also disturb the homeostatic balance of the immune response, suggesting a causal link between environmental stress and dry eye's pathogenic immune response.^26^^–^^28^

Various environments, such as air travel and offices, can potentially expose people to dry environments for long periods of time as a result of heating and air conditioning systems that dehumidify indoor climates. To improve and optimize these artificial environmental conditions, it is important to examine the effect that exposure to such climates might have on visual quality.

Blink rate and tear film osmolarity are tests that characterize different aspects of the ocular surface. An increased blink rate is usually associated with ocular discomfort, particularly in contact lens wearers.^29^^,^^30^ It has been shown that people with dry eye symptoms tend to blink more after exposure to low humidity environments,^31^ and they also show incomplete blinking.^32^ Tear osmolarity has also been postulated to be an important parameter for potential diagnosis of DED^33^ and it may also be one of its initiating factors.^34^ The Tear Film and Ocular Surface Society (TFOS) Dry Eye Workshop (DEWS) II^35^ recommended a value over 308 osmolarity (mOsm/L) as a good indicator for a deteriorated tear film.

In this study, we compare the three tests (light scattering, blinking, and osmolarity) in younger and older participants before and after the exposure to a low humidity environment.

## Materials and Methods

The study was conducted in accordance with the tenets of the Declaration of Helsinki and all procedures were approved by the research ethics committee of the Faculty of Medical Sciences (Anglia Ruskin University). Following an explanation of the nature and possible consequences of the study, informed written consent for the enrollment of 36 subjects was obtained. Subjects were clustered in two groups: younger (<40 years old, *N* = 13, 27 ± 6 years) or older (60+ years old, *N* = 23, 71 ± 7 years). Exclusion criteria included a history of ocular surgery or disease and a clinical diagnosis of cataracts or any DED.

Environmental stress was created using Anglia Ruskin's controlled environmental chamber (CEC; Weiss-Gallenkamp Ltd., Loughborough, UK). The chamber is a room with dimensions of 2.1 × 3.1 × 2 m, and the capacity to maintain a set temperature between the range of 5°C and 40°C, and relative humidity between 5% and 85%.

Initially, the humidity in the chamber was set to 45% (23°C). The participants sat in the chamber for a 15-minute equilibration period, after which measurements were performed in the following order: blinking ratio, intraocular scattering, and tear film osmolarity. After the tests were completed, the chamber was set to 5% RH and 23°C. Subjects then watched a movie (for 1.5 hours) and measurements were repeated in the same order as before.

### Intraocular Scattering

**Figure 1.**
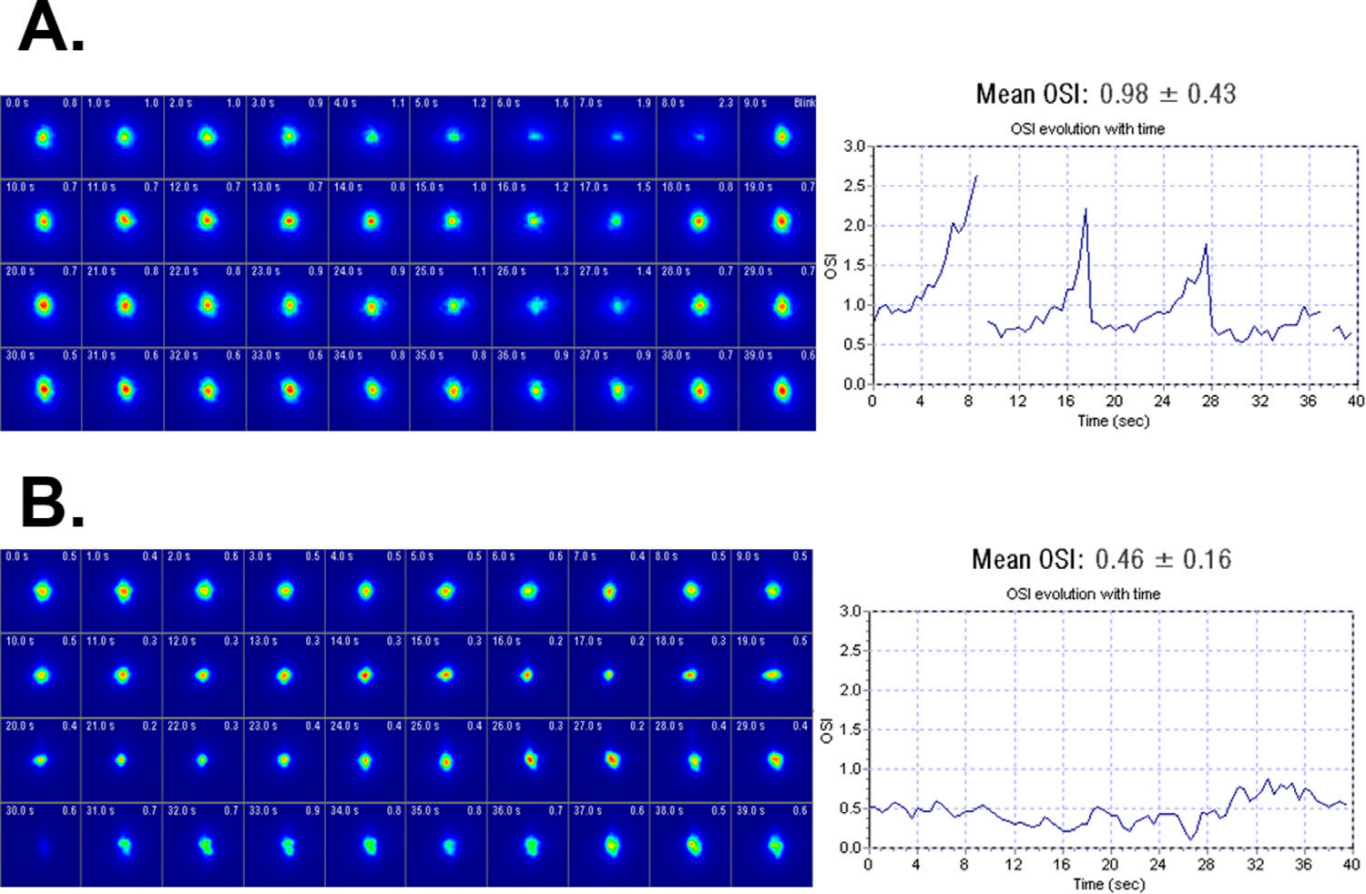
Double pass images (*left panel*) and OSI (*right panel*) as a function of time for two subjects recruited for this study. Panel (**A**) represents data for a 50 years old subject, whereas panel (**B**) was an 18 year old subject.

Intraocular scattering was measured using the Optical Quality Analysis System II (OQAS II; Visiometrics S.L., Tarrasa, Spain). OQAS II is double-pass instrument based on unequal pupil configuration with an entrance pupil diameter of 2.0 mm and an exit pupil of variable diameter, explained in detail elsewhere.^11^^,^^36^ Both ocular aberrations and scattering affect double-pass images.^36^ Intraocular scattering was quantified from the OQAS double pass images using the Objective Scatter Index (OSI), which is defined as the ratio between the integrated light in the periphery and the central peak of the DP image.^11^ The central peak zone is defined as a circle subtending a radius of 1 minute of arc, whereas the peripheral zone is a ring subtending from 12 to 20 minutes of arc. All images were acquired at best focus using a Badal optometer positioned within the instrument to correct spherical defocus (from −8 D to +6 D). Figure 1 shows two different measurements of ocular scattering. The double pass image is plotted as a function of time, for two different subjects (Figs. 1A, 1B) with very different results. In subject A, the double pass image became diffused quickly indicating that more energy was distributed fast towards the peripheral areas of the PSFs (i.e. more scattering). After blinking, most of the energy is redirected into the central areas of the PSF and then the cycle began again. This cyclic behavior generated the saw-tooth type plot (OSI as a function of time) shown on the right-hand side of the graph. On the other hand, subject B shows a very stable PSF with time and most of the energy remained into the central areas of the PSF (less scattering).

### Blinking Ratio

**Figure 2.**
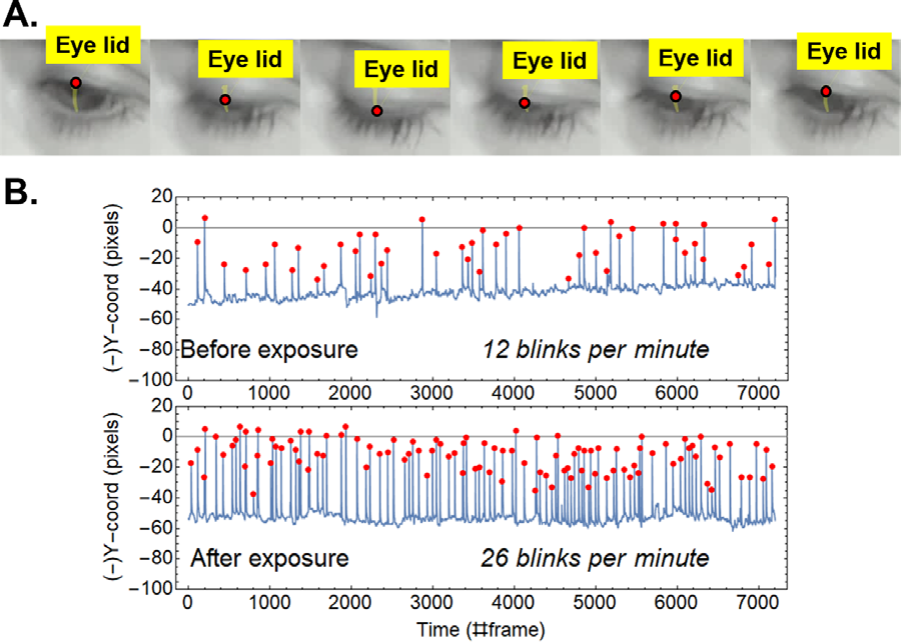
Panel (**A**) is the temporal sequence of a blink showing the routine to detect the position of the superior eye lid (*red dot* on the image). Panel (**B**) is the blinking data for one of the participants. The Y-axis corresponds to the vertical coordinate when tracking the upper lid in the eye. The X-axis represents time, corresponding to each videoframe. Video capture was performed at a rate of 30 frames per second. The interval covered in this figure correspond to a 4-minute video (7200 frames). Peaks in the figure (marked as *dots*) correspond to blinks. Upper graph and lower graphs represent data taken before and after the exposure to low humidity.

Blinking ratio was measured using an infrared video camera (Pi Noir) connected to a Raspberry Pi computer. Images were acquired at a frame rate of 30 Hz and a resolution of 800 × 600 pixels. An array of infrared LEDs (860 nm) was used to illuminate the eye. Subjects watched a 5-minute film on a TV screen. The head was stabilized using a chin/head rest. The device was placed 30 cm away from the eye subtending a 45 degree angle or higher over the line of sight, and it did not interfere with vision. Kinovea software (Kinovea 0.8.26 for Windows; available at http://www.kinovea.org) was programmed to track the movement of the upper lid during the 5 minutes of the video. Readings from the first minute were discarded to get more stable and homogenous values. Blinking was measured over the remaining 4 minutes. Mathematica software was used to automatically detect and count the blinks. Each blink corresponded to a peak that showed up when the vertical position of the lid was plotted as a function of time. Figure 2 shows an example of the imaging technique to measure blinking. In Figure 2A, a sequence of a particular blink is shown. The superior eye lid was tracked in each of the video frames. Plotting the vertical location of the eye lid as a function of time (frame number) shows each blink as represented by a peak as shown in Figure 2B. The frequency of blinks after the exposure to low humidity was significantly higher than before exposure to environmental stress.

### Osmolarity

Tear film osmolarity was measured using the TearLab system (TearLab Corp., San Diego, CA, USA). The working principle is based on calculation of the electrical impedance of a 50 nanoliter (nL) sample collected from the temporal meniscus of the tear film. The TearLab was calibrated with both the electronic check card and high control solution according to manufacturer instructions, with calibration performed on the days of testing. Data were collected as specified by the manufacturer. Subjects were requested to blink three times and look superiorly and contralaterally as instructed. The microchip collection tip of the osmolarity test card was gently placed into the tear meniscus at the lateral canthus of the eye being examined. Care was taken to make no contact with the ocular surface to avoid reflex tearing. Data were collected from a single eye per patient.

### Statistics

A mixed between-within subjects’ analysis of variance (ANOVA) was conducted to assess the impact of age (younger <40 years and older >60 years) on light scattering, blinking, and osmolarity data before and after the exposure to low humidity conditions. The independent variables were “age” (between-subjects factor) and “exposure to low humidity” (within-subjects factor). The dependent variables were OSI, blink ratio, and osmolarity. The mixed ANOVA analysis was conducted separately for each one of these dependent variables. For each analysis, we reported two “main effects” (i.e. the effect of a single independent variable on a dependent variable ignoring all other independent variables) and an “interaction effect” (if there is an interaction between the independent variables that affect the dependent variable).

Additionally, Pearson correlation coefficients between the dependent variables (OSI, blink ratio, and osmolarity) were calculated to check potential associations between them.

## Results

### Objective Scatter Index

**Figure 3.**
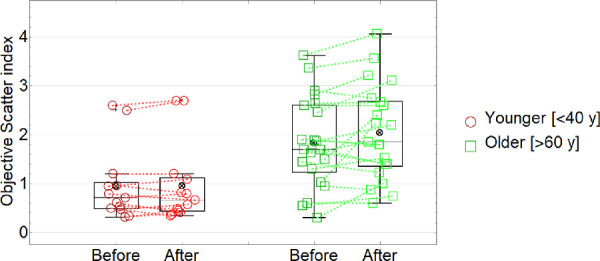
Objective scatter index (OSI) before and after the exposure to low humidity for younger and older subjects.

Figure 3 and Table 1 summarizes all OSI values measured for the younger (red circles) and older (green squares) groups. The dashed lined connects data measured before and after the exposure to 90 minutes of low humidity conditions for each subject. Black crossed circles represent the mean values in each condition. The ANOVA (Table 2) revealed a main effect of the “age” on light scattering (*F*(1, 31) = 22.02, *P* < 0.0001). The mean OSI value (including all values pre- and post-exposure) in the older group was 1.95 ± 0.93 OSI units, whereas for the younger group it was 0.67 ± 0.27 OSI units. There was also a significant main effect of the exposure to low humidity on light scattering (*F*(1, 31) = 5.45, *P* = 0.03), and a significant interaction between “age” and the “exposure to low humidity” (*F*(1, 31) = 5.12, *P* = 0.03). On average, for the older group, the values of light scatter before and after the exposure to the low humidity environment were 1.85 ± 0.92 OSI units and 2.05 ± 0.96 OSI units, respectively (*P* = 0.03). For the younger group, the values were 0.66 ± 0.27 OSI units and 0.67 ± 0.28 OSI units, respectively (*P* = 0.90), demonstrating that OSI values did not change in the younger group after exposure to low humidity but increased significantly in the older group. On average, for the older group, the increase in light scattering after the exposure to the low humidity environment was 0.2 OSI units.

### Blinking

**Figure 4.**
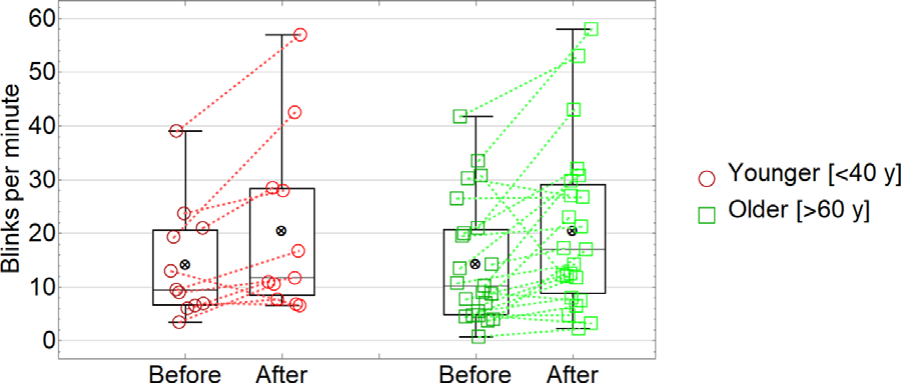
Blinking rate (blinks per minute) before and after the exposure to low humidity for younger and older subjects.

Figure 4 and Table 1 summarizes the blinking rate (blink per minute) results (red and green data represented younger and older subjects, respectively; dashed lines connect data measured before and after the exposure to low humidity for each subject). ANOVA (Table 2) revealed a main effect of the exposure to low humidity, *F*(1, 28) = 12.99, *P* = 0.001, demonstrating a higher blinking rate after the exposure to low humidity (20 ± 15 blinks/minute) compared to the baseline conditions (14 ± 11 blink/minute). There was no significant main effect of aging on blinking (*F*(1, 28) = 0.03, *P* = 0.86), with participants showing similar blinking rates for younger (17 ± 13 blinks/minute) and older (18 ± 14 blinks/minute) groups. There was also no significant interaction (*F*(1, 28) = 0.80, *P* = 0.37).

### Tear Osmolarity

**Figure 5.**
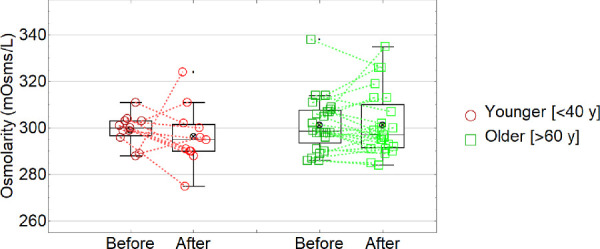
Tear osmolarity (mOsms/L) before and after the exposure to low humidity for younger and older subjects.

Figure 5 and Table 1 shows osmolarity data (red and green data represented younger and older subjects, respectively; dashed lines connect data measured before and after the exposure to low humidity for each subject). ANOVA (Table 2) showed no significant main effects of ageing on osmolarity (*F*(1, 27) = 1.23, *P* = 0.28), or exposure to low humidity (*F*(1, 27) = 0.13, *P* = 0.72) or any significant interaction effects (*F*(1, 27) = 0.0009, *P* = 0.98).

## Discussion

Light scattering is typically greater in eyes with mild to moderate DED.^16^^,^^17^^,^^37^^–^^39^ Our data also show that older subjects had higher baseline scatter than the younger group. Various differences in ocular media, including subclinical lens changes, would explain this ageing effect.^1^^,^^40^^–^^44^

We show that environmental stress, caused by low humidity, can also influence light scattering in older subjects. It is possible that the dry environmental conditions induced changes in the anterior ocular media, especially the tear film in older subjects. It is known that the tear film is sensitive to changes in relative humidity (Wolkoff 2018).^45^ We show that these changes in the tear film also manifests as light scatter. Indeed, Koh et al. suggest that light scattering is altered when viscosity and hydration of the tear film change.^46^ The simulated dry environment would have altered the tear film resulting in an increased light scatter.

It has been postulated that light scatter in patients with intraocular lenses are possibly due to small subsurface nanoglistenings of diameters less than a micron.^47^^,^^48^ It has also been shown that similar size nano-irregularities are also present in the tear film as aggregates of surface-active excipients, like dead opaque superficial epithelial cells. The presence of these opacities significantly increased in patients with dysfunctional tear syndrome.^49^ We therefore hypothesize that the density of these nano-aggregates might be higher in older subjects than in younger subjects, and that light scatter from a regular, smooth ocular surface in younger subjects would be less affected by environment stress than scatter from an uneven, irregular surface with micro/nano opacities of older participants. In the case of the uneven irregular surface, it is likely that the effect will be exacerbated resulting in a thinner, more dehydrated tear film. A recent study suggested that the presence of these imperfections in the ocular surface are associated with active interactions between certain ophthalmic drugs and surface active ophthalmic excipients,^50^ and they have been also linked to the formation of aggregates resulting from molecular interactions between lipids and proteins in the tear film layers.^51^

We demonstrate that blinking is significantly influenced by environmental stress. After 90 minutes of exposure, the blinking rate increased by a factor of 40% in both groups. Blinking protects the eyes and also increases hydration to the ocular surface. Our results agree with a previous study^52^ in younger and older subjects exposed to different levels of humidity (10%, 30%, and 50%) for 90 and 180 minutes. Although we did not extend to 180 minutes, the previous study suggested that after 90 minutes the blink rate stabilized.^52^

We found no correlation between light scatter and blink rate (Table 3). This is perhaps not surprising due to the different underlying processes. Whereas light scatter is a measure of the optical quality, blinking is a mechanical response to corneal dehydration, regardless of the optical quality of the central ocular surface. Therefore, whereas both younger and older subjects blinked more and to an equal extent to minimize corneal dehydration, the light scatter only increased in elder people, possibly due to potential differences in the quality of the ocular surface /tear film, as explained in previous paragraphs.

We found no statistically significant changes in osmolarity after the exposure to low humidity conditions after 90 minutes. Previous studies have also shown no changes in osmolarity after 60 minutes of exposure to 5% RH and 21°C,^23^ and also after a longer exposure of 120 minutes to 5% RH and 23°C.^24^ There was substantial individual variability among subjects, but the direction of the changes was inconsistent. It is possible that significant variability might be intrinsic to these kind of measurements.^53^^,^^54^ There was also no difference in osmolarity with age, as shown by previous studies.^55^^–^^57^ The baseline values of osmolarity were well within the ranges found for healthy subjects in previous studies.^35^ No correlations were found between tear osmolarity and any of the other two parameters measured in this study (Table 3). Again, this was probably due to the different physical nature of the three metrics, each one accounting for a different property of the ocular surface. Osmolarity characterized the tear film using a molecular parameter, light scatter characterized the optical quality in the center of the ocular surface, and blinking was a mechanical response against low humidity. It might also be that the increase in the number of blinks, perhaps caused by an increased reflex tearing, prevented tear osmolarity to increase after the exposure to low humidity, which might also explain why no significant correlations were shown.

There are some limitations in this study that could be addressed in future research. First, it would have been useful to include a group not exposed to low humidity to act as a control. In designing our study, the younger group was the control group in order to examine age comparisons. In addition, the sample size for the younger group (*N* = 13) was smaller than for the older group (*N* = 23). Unequal sample sizes for different cohorts are not that uncommon. However, to check for any potential overestimations of the *P* value, we developed a bootstrapping method to randomly resample the larger group of same sample size as the smaller group. We created 1000 random samples for *N* = 13 and derived significance values (ANOVA *P* value) for the interaction effect between humidity and age in the OSI value for each resample. The *P* values’ distribution showed a maximum peak between 0 and 0.05 (319 of the 1000 resamples). Because the *P* value distribution showed its maximum peak for less than *P* = 0.05, it confirmed the originally derived significant interaction effects that were reported.

Our findings show an absolute mean increase in light scatter for the older group of 0.2 OSI units. Although it is a relatively small value, it is 10% of the total OSI value reflecting to the increase of 100% in the magnitude of light scatter after exposure to the environmental stress. Whereas our study did not examine the impact of this increase of on visual function, Bueno et al.^20^ reported that an increase in one OSI unit corresponds to a drop of 0.9 units in contrast sensitivity at 12 c/deg. It should also be noted that the exposure time to environmental stress in our study was modest (90 minutes), and it is likely that people would spend longer time in environments with air conditioning (A/C) or heating units resulting in exacerbated effects. Indeed, this may be more important in people with DED and needs further investigation.

**Table 1.** Summary of the Effects of the Exposure to Low Humidity on OSI, Blink Ratio, and Osmolarity.

**OSI**						
Exposure to Low Humidity						
	Before		After		Marginal	
Age	***M***	***SD***	***M***	***SD***	***M***	***SD***
<40 y	0.66	0.27	0.67	0.28	0.67	0.27
>60 y	1.85	0.92	2.05	0.96	1.95	0.93
Marginal	1.42	0.94	1.55	1.02		
**Blink Ratio** (Blinks/Minute)						
Exposure to Low Humidity						
	Before		After		Marginal	
Age	***M***	***SD***	***M***	***SD***	***M***	***SD***

<40 y	14	10	20	16	17	14
>60 y	14	12	21	15	18	14
Marginal	14	11	21	15		
**Osmolarity** (mOsms/L)						
Exposure to low humidity						
	Before		After		Marginal	
Age	***M***	***SD***	***M***	***SD***	***M***	***SD***

<40 y	299	7	298	11	299	9
>60 y	303	12	303	14	303	13
Marginal	302	10	301	13		

M, mean value; SD, standard deviation.

**Table 2.** Mixed Between-Within Subjects ANOVA Summary Table

	OSI		Blink Ratio		Osmolarity	
	*F*	*P* Value	*F*	*P* Value	*F*	*P* Value
Age	22.0186	0.001^**^	0.0307	0.8623 NS	1.2325	0.2767 NS
Exposure to low humidity	5.45232	0.0262^*^	12.9907	0.0012^**^	0.1327	0.7184 NS
Age^*^ exposure	5.1221	0.0308^*^	0.8054	0.3771 NS	0.0009	0.9760 NS

* *P <* .05

** *P <* .01

NS, not significant.

**Table 3.** Correlation Matrix Summary Table

Correlation Matrix				
		OSI	Blink/Min	Osmolarity (mOsms/L)
**OSI**	Pearson's r	—		
	*P* value	—		
**Blink/min**	Pearson's r	−0.098	—	
	*P* value	0.455	—	
**OSMOLARITY (mOsms/L)**	Pearson's r	−0.079	0.214	—
	*P* value	0.558	0.127	—
